# Liver cirrhosis: molecular mechanisms and therapeutic interventions

**DOI:** 10.1002/mco2.721

**Published:** 2024-09-17

**Authors:** Zihe Dong, Yeying Wang, Weilin Jin

**Affiliations:** ^1^ The First School of Clinical Medicine Lanzhou University Lanzhou People's Republic of China; ^2^ Institute of Cancer Neuroscience Medical Frontier Innovation Research Center The First Hospital of Lanzhou University Lanzhou People's Republic of China

**Keywords:** drug delivery system, fibrosis, liver cirrhosis, nanomedicine, therapeutics

## Abstract

Liver cirrhosis is the end‐stage of chronic liver disease, characterized by inflammation, necrosis, advanced fibrosis, and regenerative nodule formation. Long‐term inflammation can cause continuous damage to liver tissues and hepatocytes, along with increased vascular tone and portal hypertension. Among them, fibrosis is the necessary stage and essential feature of liver cirrhosis, and effective antifibrosis strategies are commonly considered the key to treating liver cirrhosis. Although different therapeutic strategies aimed at reversing or preventing fibrosis have been developed, the effects have not be more satisfactory. In this review, we discussed abnormal changes in the liver microenvironment that contribute to the progression of liver cirrhosis and highlighted the importance of recent therapeutic strategies, including lifestyle improvement, small molecular agents, traditional Chinese medicine, stem cells, extracellular vesicles, and gut remediation, that regulate liver fibrosis and liver cirrhosis. Meanwhile, therapeutic strategies for nanoparticles are discussed, as are their possible underlying broad application and prospects for ameliorating liver cirrhosis. Finally, we also reviewed the major challenges and opportunities of nanomedicine‒biological environment interactions. We hope this review will provide insights into the pathogenesis and molecular mechanisms of liver cirrhosis, thus facilitating new methods, drug discovery, and better treatment of liver cirrhosis.

## Graphical abstract

This review emphasized the recent applications of different therapeutic strategies that regulate liver fibrosis and liver cirrhosis and highlighted the importance of nanomedicine with an emerging therapist in liver cirrhosis.

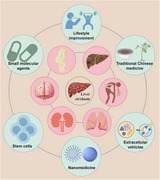

## 1 INTRODUCTION

Liver cirrhosis develops after long‐term and repeated insults to the hepatic parenchyma due to inflammation and necrosis, which results in the replacement of the healthy liver parenchyma with fibrotic tissue and regenerative nodules.^1^ Along with the disruption of hepatic architecture and vascular remodeling, liver function is impaired, the vascular tone is increased, and finally lead to portal hypertension (PH).^1^, ^2^ Because of the strong compensatory function of the liver, even early liver cirrhosis usually demonstrates no obvious clinical signs and symptoms. Subsequently, the main manifestations of liver cirrhosis appear gradually, including jaundice, spider nevus, liver palms, muscle spasms, itching, poor sleep quality, and so on.^3^ Liver cirrhosis is widely prevalent worldwide and can be a consequence of different causes, such as hepatitis B virus (HBV) or hepatitis C virus (HCV) infection, high alcohol consumption, autoimmune diseases, or cholestatic diseases.^1^, ^3^ Liver cirrhosis has been an important cause of morbidity and mortality among patients with chronic liver disease. The estimated number shows that the cases of liver cirrhosis worldwide reached 1602.43 million,^4^ and the deaths associated with liver cirrhosis worldwide was nearly 1.5 million in 2019.^3^ Therefore, it is urgent to develop a treatment method for liver cirrhosis to alleviate the social and economic burden.

Clinically, liver cirrhosis presents in two main clinical stages: compensated and decompensated (percent consensus definition, decompensation is defined by the development of clinically overt complications of PH, specifically overt ascites, variceal bleeding, or overt hepatic encephalopathy).^2^, ^5^ For the treatment of liver cirrhosis, etiological therapy, and lifestyle changes are considered for patients with compensated cirrhosis, aiming to reduce the risk of hepatic decompensation. The treatment of decompensated cirrhosis is directed at each specific complication, including ascites, variceal bleeding, and hepatic encephalopathy, which frequently presents in combination with others.^1^, ^2^, ^5^, ^6^ Additionally, liver transplantation is the most effective treatment for end‐stage liver disease, and patients with decompensated cirrhosis should be considered for liver transplantation.^2^ The idea of recompensation was proposed for individuals with decompensated cirrhosis who may experience an improvement in liver function if the etiology is removed.^2^, ^7^ The key objective of recompensation is to preserve residual liver function, slow the progression of the disease, prevent the occurrence of complications, improve patient quality of life, and simultaneously extend the waiting time for patients who may require liver transplantation.^8^ However, the impact of etiological therapy other than alcohol abstinence and antiviral treatment on recompensation still needs to be further confirmed.^2^ Fibrosis is the necessary stage and essential feature of liver cirrhosis, and effective antifibrosis strategies are therefore commonly considered the key to treating liver cirrhosis.^1^ Recently, although many different pharmacotherapeutic strategies aimed at reversing or preventing liver fibrosis progression have been proposed, the effect of these therapies is not satisfactory.^9^ This may be associated with an unclear understanding of pathogenesis, no accurate and validated drug positioning mark, limited lesion accumulation, and so on.

At present, some emerging treatment strategies for liver cirrhosis are gradually developing; in addition to small molecular agents and traditional Chinese medicine (TCM), there are also excellent treatment methods such as stem cells, extracellular vesicles (EVs), gut remediation, etc. It has shown that the latter has unique advantages in promoting liver regeneration and repair, delivering bioactive molecules and intercellular communication, and adjusting the balance of gut microbiota and metabolic products.^10^, ^11^, ^12^ Besides, nanomedicine, which used the rational application of nanoscale or nanostructured materials for delivering drugs precisely to liver tissues and enhancing the therapeutic effects of liver diseases, providing a new approach to improving the prognosis of patients with liver cirrhosis. Diverse materials consisting of different components and structures can produce nanoparticles (NPs) with specific characteristics and are tailored to localize selective tissues through conjugation to ligands that bind cell‐specific receptors.^13^, ^14^ NPs are the particularly promising drug delivery platform, which possesses the superiority that easily modulated intrinsic properties through controlling chemical synthesis, greatly improved delivery efficiency through overcoming the biological barrier to reach the lesion area, the excellent properties of biocompatibility, and minimal immunogenicity and side effects.^13^, ^15^, ^16^ Based on the current limitations of therapeutic methods aimed at reversing liver cirrhosis, these strategies may offer potential benefits.

Therefore, we summarized in detail the functions and fates of different cells in the liver microenvironment and the latest advances in therapeutic interventions for liver fibrosis and liver cirrhosis and highlighted the importance of nanomedicine with an emerging therapist in liver cirrhosis. Meanwhile, deeply understanding the interactions between nanomedicine and the biological environment is also vital to designing a safe and efficient nanomedical treatment scheme. We hope this review will help to improve our understanding and develop the next generation of antifibrotic agents to provide more effective treatment options and individualized treatment programs for liver cirrhosis.

## 2 MOLECULAR MECHANISMS: ESSENTIAL FOUNDATION FOR LIVER CIRRHOSIS TREATMENT

**FIGURE 1:**
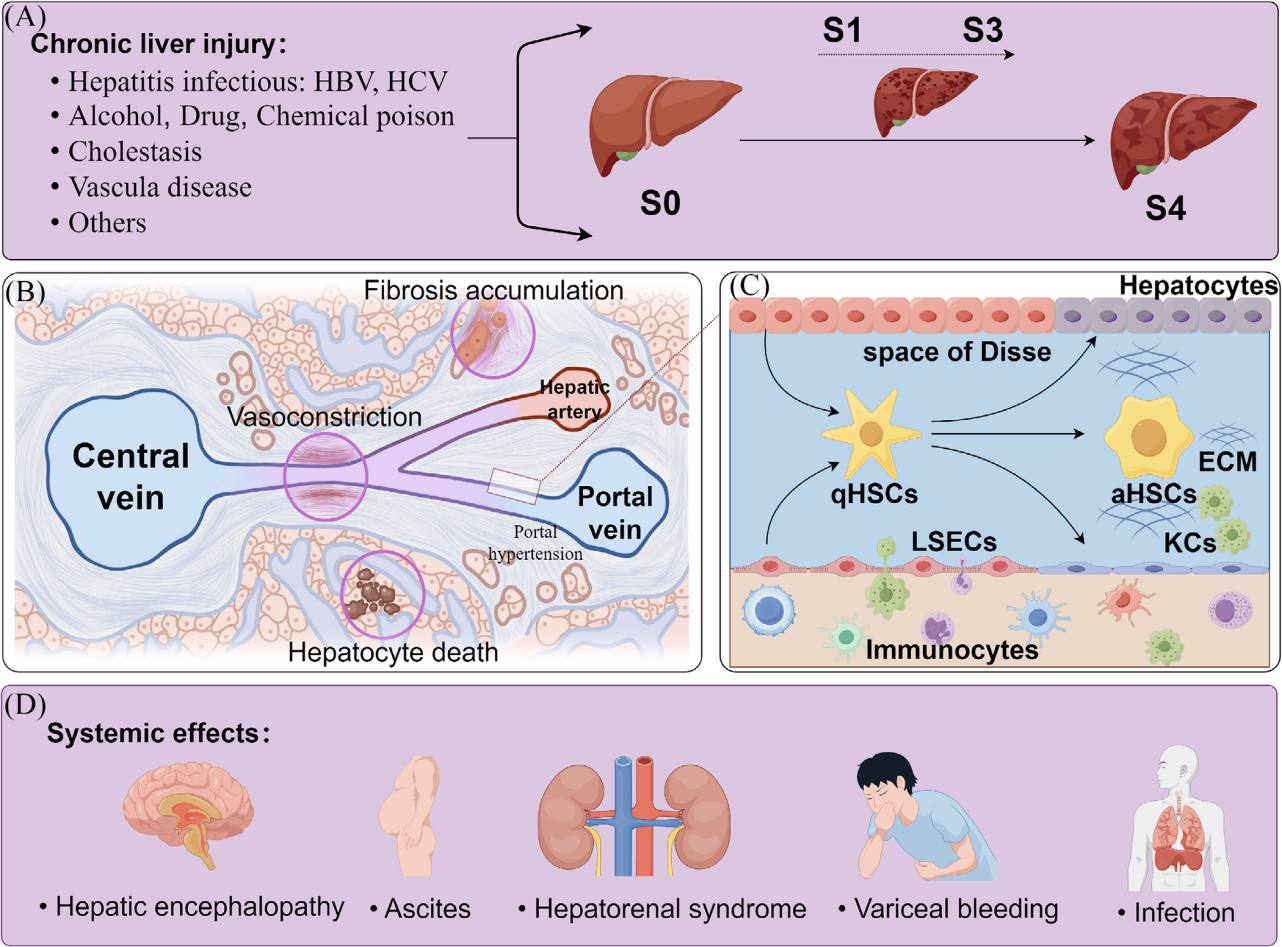
The pathophysiology and molecular mechanism of liver cirrhosis. (A) Chronic liver injury caused by various etiologies leads to liver fibrosis. Liver biopsy is the gold standard for the diagnosis of liver fibrosis, and the histopathological staging mainly includes stage 0 (S0): no definite fibrosis; stage 1 (S1): mild fibrosis; stage 2 (S2): moderate fibrosis; stage 3 (S3): advanced liver fibrosis; and stage 4 (S4): liver cirrhosis. (B) Liver cirrhosis leads to intrahepatic resistance, including hepatocyte death and replacement by fibrotic tissue, structural changes in hepatic sinuses (e.g., regenerative nodules) leading to the restriction of sinusoidal flow, and functional contraction of intrahepatic blood vessels due to the reduced production of vasodilators (e.g., NO). These features disrupt the normal metabolic functions of the liver, resulting in hepatic dysfunction, portal hypertension, and other systemic effects. (C) Long‐term inflammation leads to an imbalance in the liver microenvironment, which is associated with cell heterogeneity, interactions with various cell types, and microenvironmental factors in liver tissue and stimulates the occurrence of liver cirrhosis. (D) The systemic effects of liver cirrhosis. This figure was generated using FigDraw (https://www.figdraw.com).

The steady state of the liver microenvironment plays a crucial role in maintaining normal physiological functions of the liver, which are mostly performed by parenchymal cells, namely hepatocytes and non‐parenchymal cells (NPCs), including hepatic stellate cells (HSCs), liver sinusoidal endothelial cells (LSECs), Kupffer cells (KCs) and additional immune cell populations, as well as non‐cellular components.^17^ However, long‐term inflammation can cause continuous damage to liver tissues and hepatocytes and form a unique local microenvironment that promotes fibrosis (namely liver fibrotic niche), finally stimulating the occurrence of liver cirrhosis.^18^ These events leading to liver fibrotic niche usually involve cell heterogeneity (distinct spatial, molecular, and functional properties) and intricate interactions with various cell types and microenvironmental factors in liver tissue^19^ (Figure 1). Besides, the function of noncellular components still cannot be ignored in the liver microenvironment, maybe the essential target for liver cirrhosis treatment, such as reactive oxygen species (ROS), and the importance has been summarized in other reviews.^20^ Therefore, paying more attention to and comprehensively expounding the molecular mechanisms of liver cirrhosis and intervention strategies for multiple pathways, rather than a single pathogenic process or signal pathway, may completely stop the progress of liver fibrosis, even reverse liver cirrhosis.

### 2.1 Hepatic stellate cells

HSCs are distributed in the perisinusoidal space (space of Disse). They are surrounded by hepatocytes and LSECs, whose main functions are the secretion of laminin, proteoglycans, and type IV collagen to form basement membrane‐like structures.^21^ HSCs are the resident non‐parenchymal liver cell population and are now recognized as a remarkably plastic cell type that regulates immunity, inflammation, energy metabolism, etc.^21^, ^22^, ^23^

It is generally believed that the activation of HSCs is the primary driving factor in the occurrence and development of liver fibrosis. Under physiological conditions, HSCs contain retinoid lipid droplets and exhibit a quiescent phenotype (quiescent HSCs).^24^ When the liver is injured, quiescent HSCs are transdifferentiated into highly proliferative myofibroblasts.^23^ This activated effect can be triggered by various signals that serve as markers of cellular injury, including paracrine and autocrine loops (e.g., platelet‐derived growth factor),^24^ fibrogenic signals (e.g., transforming growth factor‐beta [TGF‐β] and connective tissue growth factor),^24^ proinflammatory cytokines (e.g., interleukin 1, interleukin 6, and tumor necrosis factor) produced by neighboring cells or complexes (e.g., inflammasomes),^21^ endothelial cell‐mediated growth factor (e.g., endothelial cells) released,^25^ ROS burden increased,^20^ etc. HSCs are the main drivers of liver fibrosis, and TGF‐β‐induced HSCs express α‐smooth muscle actin (α‐SMA), fibronectin, and type I/III collagen (Col I/II), which is the maker of active HSCs.^21^ Besides, myofibroblasts, which express abundant intracellular proteins including vimentin, α‐SMA, and non‐muscle myosin, are the primary source of secreted extracellular matrix (ECM) and generate fibrous scars in the fibrotic liver.^24^ This will lead to the distortion of liver parenchyma and vascular structure and affect the function and blood circulation of the liver.

Under certain conditions, pathologically activated HSCs in the liver microenvironment also have four fates such as transforming into quiescent HSCs,^26^ inducing programmed cell death (e.g., apoptosis,^27^ autophagy,^28^ ferroptosis^29^), mediating immune clearance (e.g., natural killer‐like T cells,^30^ γδT cells, and natural killer cells^31^) and promoting senescence.^32^ Therefore, maintaining/restoring the healthy phenotype or changing/clearing the disease‐related phenotype in HSCs is essential to prevent or relieve liver disease.

### 2.2 Liver sinusoidal endothelial cells

LSECs are the largest number of NPCs in the liver (more than 70% of NPCs),^17^ which have essential roles in the maintenance of hepatic homeostasis, including regulating immune response, maintaining vascular activity, and promoting liver regeneration.^33^, ^34^, ^35^ In the liver microenvironment, LSECs constitute the hepatic sinusoidal capillary channels and display a unique fenestrated phenotype (50‒150 nm), facilitating the direct communication between hepatocytes and oxygen, micronutrients, and macronutrients from the bloodstream.^36^ Besides, LSECs are the main source of nitric oxide (NO) in the normal liver through endothelial nitric oxide synthase (eNOS) activation by shear stress, and NO is a key vasodilator molecule regulating sinusoidal flow in the liver.^37^

Hepatic hemodynamics is the most significant effect of dysfunctional LSECs in the liver microenvironment. In response to liver injury, transcriptional co‐activator with PDZ‐binding motif (TAZ) downregulates the expression of eNOS and NO production.^38^ Subsequently, impairment of NO production results in LSEC dysfunction and affects the intrahepatic microcirculatory status, leading to microvascular dysfunction and basement membrane development (namely LSEC capillarization) and inducing liver fibrosis.^39^ Capillarized LSECs further limit macromolecular/solute exchange, causing hepatic perfusion and relative hypoxia and ultimately contributing to PH.^36^, ^39^

Moreover, LSECs modify their phenotype and negatively affect neighboring cells in the liver microenvironment, including the activation of HSCs.^36^, ^40^ LSECs participate in this process by secreting inflammatory mediators and cytokines to regulate inflammatory or immune responses. For example, LSEC‐derived adipocyte fatty acid binding protein (A‐FABP) promotes LSEC capillarization by activating Hedgehog signaling, further encouraging the expression of TGF‐β1, which perpetuates the activation of HSCs and exaggerates liver fibrosis.^40^ In another study, sphingosine 1‐phosphate receptor 2 (S1pr2) in LSECs activates the YAP signaling pathway to potentiate the transactivation of TGF‐β, which acts on HSCs in a paracrine manner and aggravated liver fibrosis.^41^

### 2.3 Hepatocytes

Hepatocytes, accounting for the largest proportion of various hepatic cells (60% of liver cells composition), are the major cell type in realizing multiple liver functions, including protein synthesis, detoxification, glucose, and lipid metabolism.^17^

Long‐term inflammation can cause continuous damage to liver tissues and hepatocytes, and dysfunctional hepatocytes release a series of signals, including damage‐associated molecular patterns (DAMPs), and promote the genesis and development of inflammation and liver fibrosis.^42^ Among them, the over‐activation of NLRP3 leads to the pyroptosis of hepatocytes and the secretion of inflammasome complexes into the extracellular space, which amplify and perpetuate inflammasome‐driven fibrogenesis.^43^ Besides, Mito‐DAMPs released from injured hepatocyte mitochondria directly activate HSCs and drive liver scarring, which is a critical determinant of the development of liver fibrosis. Still, this process is suppressed by KCs and infiltrating Gr‐1^(+)^ myeloid cells.^44^ In response to injury, hepatocytes change their gene expression and secretion profile.^24^ Among them, the crosstalk between hepatocytes and HSCs plays a key role in the progression of liver fibrosis. For example, hepatocytes significantly drive HSCs to transform from quiescent HSCs to activated HSCs by releasing TGF‐β during liver damage,^45^ and blocking the function of miRNA‐221‐3p in hepatocytes would reduce HSC activation and alleviate liver fibrosis.^46^ At the same time, aHSCs derived from bone morphogenetic protein‐1 (Bmp‐1) induce epithelial‒mesenchymal transition (EMT) of hepatocytes and promote liver fibrosis.^47^

### 2.4 Kupffer cells

KCs play a crucial role in liver immune homeostasis through regulating proinflammatory immune responses (inflammation‐associated cytokines/chemokines including CXCL10) and infiltrating immunocytes including monocytes, T cells, B cells, NK cells, etc.^48^

In recent years, it has been found that KCs are highly plastic and integrate multiple signals to shape their response, showing the polarization of inflammatory (M1) and anti‐inflammatory (M2) macrophages and rapidly changing their phenotype depending on the liver microenvironment.^49^ However, this balance will be broken when meeting liver injury, liver fibrosis, and liver cirrhosis.^50^ For example, the expression of Follistatin‐like protein 1 (FSTL1) is significantly elevated in macrophages and promotes liver fibrosis by inducing M1 polarization and inflammation based on the intracellular pyruvate kinase M2 (PKM2) reprogramming.^51^ Macrophage‐specific deficiency in Yes‐associated protein (YAP) attenuates liver fibrosis by regulating the interaction between type I interferon (IFN‐I) and endothelial cells.^52^ C‐mer tyrosine kinase (MerTK) in KCs promotes an extracellularly regulated protein kinase (ERK)/TGF‐β1 pathway that activates HSCs and induces liver fibrosis.^53^ Myeloid differentiation primary response gene 88 (MyD88) signaling in HSCs will increase the secretion of CXCL10 and promote the M1 polarization of macrophages.^54^ However, the senescence of macrophages may drive liver fibrosis regression. For instance, inhibiting the Notch signaling pathway could decrease the population of hepatic senescent cells and anti‐inflammatory macrophages, thereby facilitating liver fibrosis treatment through the enhancement of enhancer of zeste homolog 2 (EZH2)‐regulated senescence.^55^

Although macrophages derived from different sources share many similar characteristics, they also exhibit distinct and unique gene expression and functional properties. For example, the uncontrolled recruitment and activation of monocyte‐derived macrophages leads to the production of excess amounts of proinflammatory cytokines. It also accelerates the demise of hepatocytes and their differentiation and augments the progression of liver fibrosis.^56^ However, bone marrow‐derived macrophages modulate the immune microenvironment to recruit and modify the activation of endogenous macrophages and NK cells, likely leading to HSCs apoptosis and hampered fibrogenesis.^57^

### 2.5 Other cell types

There are also many other important cells in the liver microenvironment, and studying these important cells may potentially affect liver cirrhosis treatment. In addition to resident immune cell populations that remain in the liver for a long time, such as KCs, many circulating immune cell populations temporarily stay in the hepatic sinus or liver parenchyma, including natural killer (NK) cells, T lymphocytes, B lymphocytes, dendritic cells, recruited mononuclear macrophages, neutrophils, adaptive immune cells, etc.^10^, ^58^ The interaction between immunocytes and immunocytes or nonimmune cells and the mutual regulation between various subsets are diverse and complicated. For example, it is demonstrated that cholangiocytes can attract B cells to fibrotic areas through the CXCL12‒CXCR4 axis.^59^ Besides, previous studies have confirmed that immunocytes and their various subgroups can regulate the progression of liver fibrosis by influencing the activities of HSCs and generating different cell factors.^60^, ^61^ Immunocytes express different effects in response to different etiologies and stages of the disease. For example, Tregs play an active role in modulating effectors of immune response to HBV infection, but they can also form immunosuppression and provide chances for HBV infection.^62^ The interaction mechanisms of most immunocytes have not been explored, especially the newly emerged subpopulations that have been identified by single‐cell technologies.

## 3 POTENTIAL THERAPEUTIC STRATEGIES FOR LIVER CIRRHOSIS

Comprehensive therapy is the best approach for managing liver cirrhosis, which adopts various treatment methods, including lifestyle improvement, etiological therapy, medication, liver transplantation, gut remediation, stem cells, and EVs therapy, to delay the progress of liver cirrhosis, relieve symptoms, and improve the survival rate and quality of life.^1^, ^2^ Etiological therapy and liver transplantation have been reviewed in detail elsewhere,^63^, ^64^ followed by more in‐depth discussions of other methods as they apply to the unique advantages.

### 3.1 Lifestyle improvement

An increasing amount of clinical evidence suggests a close relationship between chronic liver diseases (i.e., liver cirrhosis) and lifestyle habits.^65^, ^66^ Healthy food (i.e., Mediterranean diet) contains anti‐inflammatory, antioxidative, and antifibrotic components and is seemingly beneficial in managing liver diseases.^67^ Besides, coffee intake is significantly related to reducing liver fibrosis in metabolic dysfunction‐associated steatotic liver disease (MASLD). Compared with patients who did not drink coffee, drinking coffee will reduce liver fibrosis by 33%.^68^ This is closely associated with the fact that caffeine can reduce oxidative stress and liver inflammation.^66^ The beneficial effects of exercise have been evidenced in liver diseases, especially MASLD.^69^ For example, daily step counts are associated with higher hospital admission and mortality rates in patients with liver cirrhosis. Importantly, there was a 5% reduction in risk for admission and a 12% risk for death for every additional 500 steps taken per day.^70^ An animal experiment showed that exercise training would attenuate liver cirrhosis‐associated cardiac remodeling and diastolic dysfunction and prevent systolic impairment.^71^ Therefore, diet in the sense of healthy nutrition and moderate exercise should be taken based on the severity of the condition in patients with liver cirrhosis. However, the most difficult thing in life is to change habits, behaviors, and preferences, such as switching from an inflammatory to an anti‐inflammatory diet and low/no to high/real exercise.^12^

### 3.2 Small molecular agents

The recent advancements in pharmacological interventions for liver fibrosis/cirrhosis are summarized (Table 1). They mediate liver fibrosis regression of liver cirrhosis mainly through inhibiting HSC activation, regulating immune response, protecting hepatocyte function, degrading the ECM, etc.^24^ However, most clinical trials in these drugs concentrated on phase I/II. On March 14, 2024, Resmetirom, a thyroid hormone receptor‐B (THR‐B) agonist developed by Madrigal Pharmaceuticals, was approved by the Food and Drug Administration for the treatment of metabolic dysfunction‐associated steatohepatitis with liver fibrosis.^72^ This is a landmark event, but antifibrosis drugs used for liver cirrhosis treatment still need further exploration.

**TABLE 1:** Clinical trials of some small molecular agents used for liver fibrosis/cirrhosis treatment.

Drug	Targeted cell type	Signaling pathway	Disease condition	Phase	Clinical trial number
Hydronidone	HSCs	IRE1α‐ASK1‐JNK signaling pathway (↑)^73^	HBV‐related liver fibrosis	III	NCT05905172
		TGFβ‐Smad signaling pathway (↓)^74^			
Candesartan	Endothelial cells	Nostrin‐eNOS‐NO signaling pathway (↓)^75^	HCV‐related liver fibrosis; Alcoholic liver fibrosis	I, II III	NCT03770936 NCT03770936
Emricasan	Hepatocytes	Improve the phenotype of hepatocytes and their paracrine mechanisms^76^	Decompensated cirrhosis	II	NCT03205345
Selonsertib	HSCs	ASK1/MAPK signaling pathway (↓)^77^	Decompensated cirrhosis	III	NCT03053063
Cenicriviroc	Macrophages	CCR2‐STAT1/NF‐κB/ERK signaling pathways (↓)^78^	Non‐alcoholic Steatohepatitis and liver fibrosis	III	NCT03028740
	Macrophages	Depend on the phosphorylation of Erk to promote the expression of Hif1α^79^			
Resmetirom	–	STAT3 and NF‐κB signaling pathway (↓)^80^	Non‐alcoholic Steatohepatitis and liver fibrosis	III	NCT03900429
Obeticholic acid	HSCs	FXR signaling pathway (↑)^81^	Primary biliary cholangitis and liver cirrhosis	IV	NCT02308111
Pirfenidone	HSCs	Target Glutaredoxin‐1 and promote the deglutathionylation of Smad3 (↑)^82^ TGF‐β/Smad signaling pathway (↓)^83^	Advanced liver fibrosis Advanced liver fibrosis Compensated cirrhosis	II II II	NCT04099407 NCT05542615 NCT06267794
Atorvastatin	HSCs	The expression of NOX1, Rac1‐GTP, and Rac1 (↓); oxidative stress (↓)^84^	Liver cirrhosis	IV	NCT04072601
Atorvastatin coenzyme A	–	Non‐canonical Hh signaling pathway (↓)^85^	Liver cirrhosis	IV	NCT04072601

*Note*: “↑” represents the promotion of the signaling pathway and “↓” represents the inhibition of the signaling pathway.

Abbreviations: ASK1, apoptosis signal‐regulating kinase‐1; CCR2, CC chemokine receptor 2; eNOS, endothelial nitric oxide synthase; FXR, farnesoid X receptor; HBV, hepatitis B virus; HCV, hepatitis C virus; Hh, hedgehog; Hif1α, hypoxia‐inducible factor 1alpha; HSCs, hepatic stellate cells; IRE1α, inositol‐requiring enzyme‐1alpha; JNK, c‐Jun N‐terminal kinase; MAPK, mitogen‐activated protein kinase; NO, nitric oxide; NOX1, NADPH oxidase 1; STAT1, signal transducer and activator of transcription 1; STAT3, signal transducer and activator of transcription 3; TGF‐β, transforming growth factor‐beta.

*Data sources*: PubMed (https://pubmed.ncbi.nlm.nih.gov/) and Clinicaltrial (https://clinicaltrials.gov/).

At present, significant challenges remain in developing small molecule drugs that treat liver cirrhosis, mainly due to the difficulty of finding suitable binding sites on the protein‒protein interaction surfaces. And it is also increasingly difficult to screen new targets due to long‐term development and utilization. Therefore, future research needs to focus on applying innovative technology (i.e., nanotechnology or artificial intelligence) to accelerate the drug screening and design process. Besides, the complicated pathological mechanism of liver cirrhosis, drugs developed for a single target are difficult to be effective in clinical practice. Potential combination therapies with synergistic effects are needed to pursue more precise personalized drug combinations.

### 3.3 Traditional Chinese medicine

TCM, which involves syndrome differentiation and treatment, holistic effects, and multi‐target pharmacological effects, is significantly effective in treating various liver diseases, including liver cirrhosis. Some compound prescriptions of TCM, such as Fuzheng Huayu (FZHY) Formula, Fufang Biejia Ruangan (BR) Tablets, and Anluo Huaxian (ALHX) Pills, have been proven to have excellent antifibrosis effects (Table 2). For example, on the HBV‐associated liver cirrhosis patients who received 2 years of entecavir (ETV) but still with advanced fibrosis, data show that continued scar regression in 56.06% of liver cirrhosis throughout FZHY application for 48 weeks as assessed by liver biopsy.^86^ Besides, a randomized controlled trial was conducted involving 780 newly diagnosed patients with chronic hepatitis B (CHB), which shown the early combination of ALHX could further reverse liver fibrosis in CHB patients. After 78 weeks of treatment, the fibrosis regression rate and liver stiffness measurement decreased in the ETV + ALHX group, which was significantly higher than that of the ETV group at baseline *F* ≥3 patients.^87^ Another study also showed the effectiveness of BR in reversing liver fibrosis and cirrhosis. In the per‐protocol (PP) analysis of 705 patients, the ETV + BR group had a significantly higher rate of fibrosis regression than the control group. In the PP analysis of 388 patients with liver cirrhosis at baseline, the ETV + BR group had a significantly higher rate of cirrhosis regression than the control group.^88^ Similarly, TCMs can also relieve symptoms such as fatigue and loss of appetite in the liver cirrhosis treatment, thus improving the quality of life, such as Ginseng and Angelica sinensis. These essential values still need to be verified and explained.

**TABLE 2:** Summary of some traditional Chinese medicine used for liver fibrosis/cirrhosis.

Traditional Chinese medicine	Composition	Animal model	Mechanism	References
Fuzheng Huayu Formula	Taoren, Dongchongxiacao, Jiaogulan, Danshen, Songhuafen, Wuweizi	CCl_4_	Suppress hepatocyte apoptosis through regulating mediators in death receptor and mitochondrial pathways	^92^
		CCl_4_	Regulate the recruitment and polarization of intrahepatic macrophages via CCL2 and CX3CL1, and play the anti‐inflammation and antifibrosis pharmacological effects	^93^
Fufang Biejia Ruangan Tablets	Biejia, Ezhu, Chishao, Danggui, Sanqi, Dangshen, Huangqi, Ziheche, Dongchongxiacao, Banlangen, Lianqiao	CCl_4_	Eliminate oxidative stress and inflammation, and inhibit HSCs activation and ECM production by blocking TGF‐β1/Smad signaling pathway	^94^
Anluo Huaxian Pills	Sanqi, Shuizhi, Dilong, Jiangcan, Baishu, Yujin, Niuhuang, Dahuang, Shuiniujiao, Jineijin	DMN	Protect liver function and inhibit liver fibrosis in rats through increasing MMP‐2 expression	^95^
		CCl_4_	Inhibition of TGF‐β1 synthesis and TGF‐β1/Smads signaling pathway, which suppresses the activation of HSCs and reverse fibrosis	^96^
Yinchenhao Decoction	Yinchen, Zhizi, Dahuang	DMN	Regulate enzymes responsible for bile acid metabolism and inhibit HSC proliferation and activation via TGF‐β1/Smad/ERK signaling pathway	^97^
		Biliary obstructive cirrhosis	Inhibit BEC proliferation by downregulation of PDGF‐β mRNA expression, inhibit BEC profibrogenic paracrine and the epithelial‒mesenchymal transition pathological process	^98^
Xiaochaihutang	Chaihu, Huangqin, Huangqin, Banxia, Shengjiang, Dazao, Gancao	CCl_4_	Upregulation of the Nrf2 pathway against oxidative stress, making further inhibition of activated HSCs	^99^
Xiayuxue Decoction	Dahuang, Taoren, Zhechong	CCl_4_	Inhibit both HSCs activation and LSECs defenestration, which accompany chronic liver injuries and reverse the myofibroblastic HSCs into quiescent	^100^
Yiguanjian Decoction	Beishashen, Shengdi, Maidong, Danggui, Gouqizi, Chuanlianzi	CCl_4_	Enhance FLSPC‐mediated repair of liver cirrhosis through regulation of macrophage activation state	^101^
Xiaoyaosan	Chaihu, Danggui, Baishao, Baishu, Fuling, Shengjiang, Zhigancao	CCl_4_	Anti‐fibrosis effect via the TGF‐β1/Smad and Akt/FoxO signaling pathways	^102^
Danggui Shaoyao San	Danggui, Shaoyao, Zexie, Huangqin, Baizhu, Gandilong, Baijuhua, Bohe, Chuanqiong, Gancao	CCl_4_	Alleviate liver fibrosis and protect the gut barrier by affecting the gut microbiota and metabolites	^103^

Abbreviations: BEC, biliary epithelial cell; CCL2, C‒C motif chemokine ligand 2; CCl_4_, carbon tetrachloride; CX3CL1, C‒X3‒C motif chemokine ligand 1; DMN, dimethylnitrosamine; ERK, extracellular signal‐regulated kinase; FLSPC, fetal liver stem/progenitor cell; FoxO, forkhead box O; HSCs, hepatic stellate cells; LSECs, liver sinusoidal endothelial cells; Nrf2, nuclear factor erythroid 2‐related factor 2; PDGF‐β, platelet‐derived growth factor‐β; TGF‐β1, transforming growth factor‐β1.

The active components of TCM are active substances with clear molecular formulas and spatial structures separated from the compound prescription of TCMs, such as flavone, polysaccharide, saponin, alkaloid, etc. The positive therapeutic effects (i.e., inhibit fibrosis, protect hepatocytes, and promote liver regeneration) in liver cirrhosis have also been confirmed.^89^ For example, Quercetin (3,3,4,5,7‐pentahydroxyflavone, QE) improved liver fibrosis induced by BDL and CCl_4_ through attenuating HSCs activation and reducing autophagy, which regulates crosstalk between the TGF‐β1/Smads and PI3K/Akt pathways.^90^ In another study, *Lycium barbarum* polysaccharides are the major bioactive components of the *L. barbarum* (Goji), which can alleviate CCl_4_‐induced liver fibrosis by inhibiting the Toll‐like receptor (TLRs)/tenuclear factor kappa B (NF‐κB) signaling pathway and improving liver inflammation.^91^ Other active components, including Resveratrol, Oroxylin A, and Quercetin, also have the same effects.^89^

The TCMs and their active components have a potential impact on liver cirrhosis treatment. However, the application still faces some challenges and problems to be solved, such as a longer duration to treat liver cirrhosis required, variable efficacy based on the constitution, severity and adherence, lack of standardization, and limited scientific evidence, etc. In the future, the clinical application and mechanism research still need to be further explored.

### 3.4 Stem cells

Novel stem cell‐based therapeutics might be potentially important cutting‐edge approaches for treating liver diseases. Among them, mesenchymal stem cells (MSCs) are the most widely used and exist in various tissues, such as bone marrow, umbilical cord tissue, human placental tissue, adipose tissue, and menstrual blood.^104^ Stem cells not only have multidirectional differentiation potential to promote liver regeneration but have influences on liver injury resistance, immunosuppression, myofibroblast suppression, ECM degradation and remodeling, as well as reducing liver fibrosis through secreting different cytokines (interleukin‐4, 6, 10), growth factors (hepatocyte growth factor, heparin binding endothelial growth factor, connective tissue growth factor), matrix metalloproteinases (MMP‐2, 9, 13), etc.^105^ Stem cell therapy is considered an effective alternative therapy for liver diseases. Many clinical studies have proved that stem cell therapy effectively improves liver function and survival rates in patients with liver cirrhosis.^106^, ^107^ Although remarkable progress has been made in stem cell research, the safety and efficacy of stem cells in liver cirrhosis treatment are still key issues that need further evaluation. Recently, there have been some pessimistic examples of stem cell therapy. For example, compared with standard care, granulocyte colony‐stimulating factor with or without hemopoietic stem cell infusion did not improve liver dysfunction or fibrosis and may be associated with increased frequency of adverse events.^108^ This effect may be related to the dynamic interaction and paracrine between stem cells and host cells. Besides, there are still some problems, such as the low proportion of transplanted stem cells in the liver, low efficiency of transplantation, even heterotopic transplantation, and whether the delivered stem cells reach their expected site and function effectively remains to be further determined.^105^

### 3.5 Extracellular vesicles

EVs are membrane‐bound particles released by cells into the extracellular space, which are reported to be secreted ubiquitously from all domains of life, from archaea and bacteria to eukaryotes.^11^ They can be divided into three subgroups according to their diameter: exosomes (30‒150 nm), microvesicles (100‒1000 nm), and apoptotic bodies (50‒5000 nm, generated during cell apoptosis).^104^ EVs can deliver signaling substances, including DNAs, RNAs, microRNAs (miRNAs), long non‐coding RNAs, lipids, and proteins into recipient cells,^109^ which have important roles in numerous physiological (including intercellular communication, immune responses, reproduction, and development) and pathological (including tumors, metabolic diseases, and neurodegeneration) processes.^110^, ^111^

#### 3.5.1 Stem cell‐derived extracellular vesicles

An increasing number of studies show that EVs can be used as a natural therapeutic ingredient for treating a variety of common and refractory diseases, especially MSC‐derived EVs, which reportedly have therapeutic effects on liver fibrosis or liver cirrhosis.^112^ For example, Azizsoltani et al. proposed obeticholic acid (OCA)‐loaded exosomes derived from human Warton's Jelly MSCs (Exo‐OCAs) to facilitate liver recovery. Exo‐OCAs prevent the activation of HSCs by promoting the expression of the farnesoid X receptor‒cytochrome P450 7A1 (FXR‐Cyp7a1) cascade to reduce liver fibrosis. Furthermore, Exo‐OCAs promote ECM reconstruction through upregulation of MMP‐13 and downregulation of tissue inhibitor of metalloproteinase 1 (TIMP‐1).^81^ Besides, human bone mesenchymal stem cell‐derived exosomes (hBM‐MSCs‐Ex) could ameliorate CCl_4_‐induced liver fibrosis by inhibiting HSC activation through the Wnt/β‐catenin pathway.^113^ However, the EVs released by stem cells may vary depending on different environments, and this process may result from the mutual regulation between stem cells and their surrounding microenvironment.^114^, ^115^ Further exploration is needed to elucidate the specific mechanisms by which EVs exert therapeutic effects.

#### 3.5.2 Plant‐derived extracellular vesicles

Unlike stem cell‐derived EVs, plant‐derived EVs are primarily derived from edible plants, including vegetables, fruits, medicinal plants, etc.^116^, ^117^ (Table 3). Owing to their natural sources, plant‐derived EVs present several obvious advantages as therapeutic agents, such as being environmental friendly, easily accessible on a large scale, most cost‐effective in biotherapeutic applications, free of human disease‐inducing pathogens, no detectable toxicity or immunogenicity, etc.^116^ Current research has found that plant‐derived EVs play a vital role in regulating intestine microecology,^118^ suppressing inflammatory cytokines,^119^ and inhibiting mitochondrial dysfunction and oxidative stress.^120^ These effects seem closely related to regulating the pathological process of liver diseases. However, advancing clinical applications of plant‐derived EVs still need to determine further the biogenesis mechanism, better explore the stability and preservation conditions, consider all factors (plant species, status such as dry or wet, transport, seasonal, and regional factors, etc.) to choose a suitable extraction method, unifying the industry standard, and provide many preclinical studies to support the dose, safety, and efficacy of the promising drugs.^116^, ^121^

**TABLE 3:** Some plant‐derived extracellular vesicles (EVs) are used to treat liver diseases.

Source	Liver disease	Effects	References
Ginger	Alcohol‐induced liver injury	Activate the expression of liver detoxifying/antioxidant genes associated with Nrf2 and inhibit the production of ROS	^122^
Garlic	Acute liver failure	Ameliorate inflammatory eruptions, hinder the migration of circulating monocytes to the liver, and decrease macrophage infiltration	^123^
Shiitake mushroom	Fulminant liver failure	Inhibit the activation of NLRP3 inflammasome by preventing inflammasome formation in primary macrophages	^124^
Blueberry	MASLD	Inhibit mitochondrial dysfunction and oxidative stress	^120^
Asparagus cochinchinensis	HCC	Specific antitumor proliferation activity associated with the apoptosis‐inducing pathway	^125^
Cannabis	HCC	Arrest the cell cycle and induce cell death by activating mitochondrial‐dependent apoptosis signaling pathways	^126^
Grapefruit	Liver metastasis of colon cancer	Carry miRNA‐18a to induce the expression of macrophage IFN‐γ and activate NK cells and NKT cells	^127^

Abbreviations: HCC, hepatocellular carcinoma; INF, interferon; MASLD, metabolic dysfunction‐associated steatotic liver disease; miRNA, microRNA; NK, natural killer; Nrf2, nuclear factor erythroid 2‐related factor 2; ROS, reactive oxygen species.

#### 3.5.3 Microbiota‐derived extracellular vesicles

Microbiota‐derived EVs are nanosized membrane vehicles (∼20‒300 nm in diameter) composed of a lipid bilayer with various proteins and nucleic acids.^128^, ^129^ Generally, bacterial extracellular vesicles (bEVs) produced by Gram‐positive bacteria are termed cytoplasmic membrane vehicles, and they are formed through endolysins or other peptidoglycan‐damaging enzymes and treatments.^128^, ^129^ The bEVs secreted by Gram‐negative bacteria are called outer‐membrane vehicles (OMVs), which originate from two main routes: membrane blebbing and explosive cell lysis.^128^ Whether Gram‐positive or negative bacteria, it should be noted that not every bacterium produces EVs that are beneficial. Probiotics are live microorganisms that are representative of beneficial bacteria that confer a health benefit on the host.^130^ Suitable supplementation of EVs from probiotics may produce beneficial effects on liver diseases. For example, *Lactobacillus rhamnosus* GG‐derived exosomes can reduce hepatic bile acid synthesis and lipogenesis and protect against alcohol‐associated liver disease through the regulation of intestinal miRNA‐194‐farnesoid X receptor (FXR) signaling.^131^ However, some studies suggested that bEVs from intestinal flora would promote the pathogenesis of liver cirrhosis. For example, *Escherichia coli*‐derived OMVs induce inflammation and fibrosis while decreasing albumin production and affecting the prognosis of patients with decompensated cirrhosis.^132^ Therefore, inappropriate MEVs supplementation may aggravate the development of disease. For bacteria in the intestine that produce harmful EVs, inhibiting the secretion of EVs via suppressing their biogenesis pathways to reduce the delivery of toxic components or blocking EVs associated with immune activity and compounds may also be a favorable method for treating liver diseases.^128^

### 3.6 Gut remediation

Liver cirrhosis appears as the prototypic disease entity driven by the altered gut‒liver axis, characterized by an impaired gastrointestinal barrier with endotoxin and other pathogen‐associated molecular patterns overwhelming the liver.^133^ The study found that restoring and reconstituting stable intestinal microecology through diet, exercise, antibiotics, and fecal microbiota transplantation in chronic diseases can increase the chances of sustaining a healthy intestine and barrier function.^12^ Probacine could modulate gut microbiota through postbiotics (including SCFAs, proteins, peptides, hormones, and neuroactive compounds), thereby treating or delaying disease progression on the one hand, and degrade or absorb detrimental metabolites produced by diseases and harmful exogenous substances and lessen their impact on the body on the other.^130^ However, interventional trials are needed to assess the functional mechanisms and clinical outcomes associated with these therapies. Besides, the process of genomic changes in the gut microbiota related to liver cirrhosis is unclear. The association between the emergence of specific bacterial colonies and the development of liver disease and how liver cells interact with antigens from the gut are still issues that require further research.^133^ Although the changes in the intestinal flora related to liver cirrhosis are not entirely clear, the importance of intestinal flora and regulating intestinal flora in promoting the progression of liver cirrhosis can be determined.

## 4 NANOMEDICINE‐BASED DRUG DELIVERY SYSTEM IN LIVER CIRRHOSIS

**FIGURE 2:**
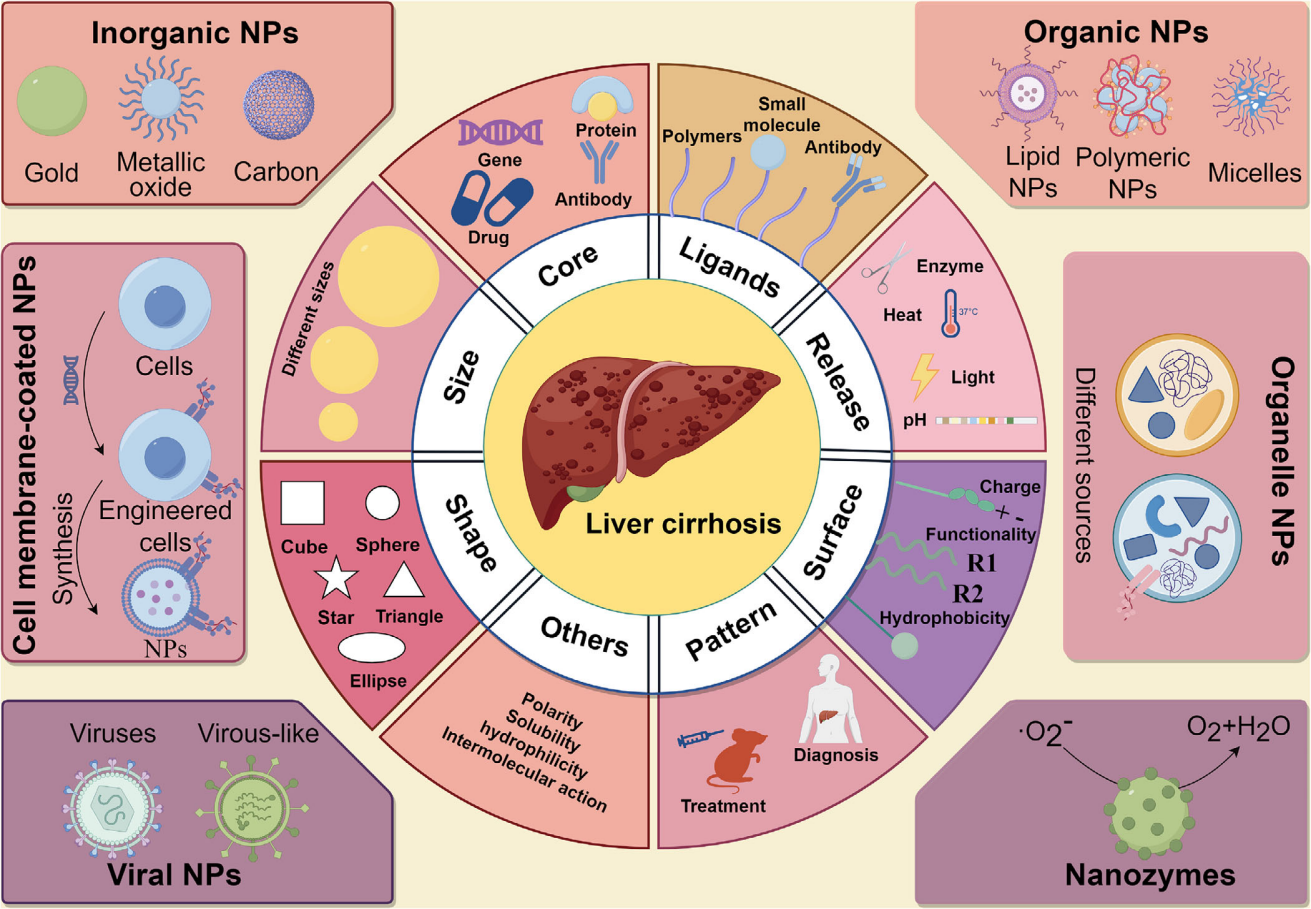
The fundamental features and different types of nanoparticles (NPs) used for liver cirrhosis treatment are described. The precise tuning of fundamental features for these NPs enables transport through biological barriers, adds biocompatibility, achieves individual treatment, etc. This figure was generated using FigDraw (https://www.figdraw.com).

NPs used for liver cirrhosis treatment can effectively improve therapeutic effects and reduce side effects, thus delaying further progress. It depends on several unique features exhibited in liver cirrhosis, which provides numerous therapeutic opportunities for NPs to treat liver cirrhosis, including (1) more easily change abnormal phenotype and function of various cell types; (2) reaction with various cytokines, chemokines, and growth factors, and influence the uptake, distribution, and release of encapsulated drugs; (3) altered ECM composition and increased density, and reduce excessive deposition; (4) increase residence time or therapeutic effects through created abnormal regions such as altered blood flow and vascular architecture can create regions of hypoxia or low perfusion; (5) enhance permeability and retention; (6) relatively preferable tolerance to foreign substances, etc.^20^, ^24^, ^134^ With the rapid development of nanomedicine, materials that can be used to design NPs are enormous, the number of NPs used in drug research is rapidly increasing, and drug research on liver fibrosis will be pushed to a new height (Figure 2).

### 4.1 Inorganic nanoparticles

Inorganic NPs have unique magnetic and optical properties, which provide multipurpose platforms for a broad range of delivery applications in drugs, proteins, and nucleic acids.^135^ Currently, inorganic NPs, which feature unique physiochemical characteristics and structural capabilities, are widely used in the research on liver cirrhosis, such as metal NPs, including Au^136^ and Se^137^ NPs, metal oxide NPs, including Fe_2_O_3_,^138^ CeO_2_,^139^ and TiO_2_ ^140^ NPs, graphite NPs,^141^ etc. No matter what kind of inorganic NPs, they have essential therapeutic effects for liver cirrhosis. For example, Tran et al. prepared sorafenib‐encapsulated siRNPs (sora@siRNPs) by self‐assembling the amphiphilic polymer PEG‐b‐siPMNT in the aqueous phase.^142^ The sorafenib released by sora@siRNPs shows antifibrotic effect through internalizating into the bloodstream and accumulating in the liver, and the remaining siRNPs in the intestine function as antioxidants and effectively scavenge the locally overproduced ROS, reducing the adverse effects of sorafenib in the gastrointestinal tract. In another study, Xu et al. developed esterase‐responsive carbon quantum dot‐dexamethasone (CD‐Dex).^143^ CDs can rapidly accumulate in the liver and target hepatocytes, efficiently preventing liver fibrosis by eliminating the intrahepatic ROS and suppressing KCs and HSCs. Meanwhile, the released Dex can suppress liver fibrosis by inhibiting the infiltration of inflammatory cells.

Inorganic NPs not only provide opportunities to reverse liver fibrosis but also provide excellent choices for imaging.^144^ For example, rare earth‐doped inorganic NPs, as the late‐model materials, have broad application prospects in the biomedical field.^145^, ^146^ Nowadays, inorganic NPs have been widely used to construct intelligent detection platforms or drug delivery systems, and the use of organic materials to modify inorganic NPs has also been proven to significantly improve the bioactivity significantly.^147^ However, some negative reports exist about the interaction between inorganic NPs and the liver. For example, iron oxide NPs can aggravate hepatic steatosis and liver injury in susceptible livers, which is associated with endoplasmic reticulum stress.^148^ Similarly, the liver is gradually considered one of the main organs in which SiO_2_ NPs accumulate, and long‐term SiO_2_ NPs exposure could induce liver dysfunction and liver fibrosis in vivo.^149^, ^150^ Therefore, while maximizing the biomedical application value of inorganic NPs, we cannot ignore their disadvantages, which may be the bottleneck for NPs in treating liver cirrhosis.

### 4.2 Organic nanoparticles

#### 4.2.1 Lipid nanoparticles

Lipid nanoparticles (LNPs) represent the most advanced system for the delivery of nucleic acids and drugs to the liver, which are typically composed of four major components: cationic or ionizable lipids that complex with negatively charged genetic material and aid endosomal escape, phospholipids for particle structure, cholesterol for stability and membrane fusion, and PEGylated lipids to improve stability and circulation.^151^, ^152^, ^153^ Nucleic acid loaded into NPs can correct the course of liver cirrhosis through gene silencing, expression, and editing, and LNPs are the widely used materials^154^, ^155^, ^156^, ^157^ (Table 4). The ionizable LNPs are ideal carriers in nucleic acid therapies because they are near neutral charges at physiological conditions. In contrast, charges in acidic inclusions can facilitate the escape to intracellular release.^153^ For example, Zhang et al. prepared pPB peptide‐modified and HMGB1‐siRNA‐loaded stable LNPs (HMGB1‐siRNA@SNALP‐pPB) to treat liver cirrhosis effectively.^156^ The LNPs were actively targeted to HSCs by the mediation of pPB peptide, effectively silenced the HMGB1 gene, inhibited the activation and proliferation of HSCs, inhibited collagen deposition and fibrosis formation in the liver, and significantly prolonged the survival time of mice models.

**TABLE 4:** Summary of some organic nanoparticles used for liver fibrosis/cirrhosis treatment.

Nano‐platform	Proprietary name	Active ingredients	Surface modification	Cell types	Target	Reference
LNPs	mLNP‐siHMGB1	HMGB1 siRNA	Mannose	Macrophages	Mannose receptors	^154^
LNPs	Cur‐mNLCs	Curcumin	PS	Macrophages	Endocytosis	^162^
LNPs	M6P‐HSA‐MT‐SLN	Matrine	Mannose 6‐phosphate	HSCs	Mannose receptors	^163^
LNPs	VA‐PFD@ZIF‐8@DMPC NPs	Pirfenidone	Vitamin A	HSCs	RBPR receptors	^164^
LNPs	AA‐T3A‐C12/siHSP47 LNPs	HSP47 siRNA	Anisamide ligands	HSCs	Sigma receptors	^155^
LNPs	VA‐SLNs	Butein	V_A_‐Myrj52	HSCs	RBPR receptors	^165^
LNPs	CT‐VLNPs	siCol1α1/siTIMP‐1	Chol‐PEG‐V_A_	HSCs	RBPR receptors	^157^
Polymer NPs	HA‐PEI/siRNA	COX‐1 siRNA	HA‐PEI	LSECs	Endocytosis	^166^
Polymer NPs	HA@PRB/COL NPs	COL	HA	HSCs	CD44 receptor	^167^
Polymer NPs	T‐C‐siRNA	PDGFR‐β siRNA	Vitamin A	HSCs	RBPR receptors	^168^
Micelles	PEG‐PCL	Camptothecin	Folic acid	HSCs	FRα receptors	^169^
Micelles	Sel@GBRNPs	Selonsertib	β‐d‐Galactose	Hepatocytes	Asialoglycoprotein receptor	^170^

Abbreviations: Cur, curcumin; FRα, folate receptor alpha; HA, hyaluronate; HMGB1, high mobility group box 1; HSCs, hepatic stellate cells; HSP47, heat shock protein 47; LNPs, lipid nanoparticles; LSECs, liver sinusoidal endothelial cells; PDGFR‐β, platelet‐derived growth factor receptor beta; PS, phosphatidylserine; RBPR, retinol‐binding protein receptor; siRNA, small interfering RNA.

Besides, they have gained significant attention due to their unique properties, such as enhanced drug delivery, controlled release of drugs, excellent biocompatibility and biodegradability, etc. For example, Zhang et al. proposed methods that interrupt the negative interactions and cellular crosstalk to break the malignant cycle and remodel the abnormal cellular network, thereby improving liver fibrosis therapy.^158^ They designed vismodegib (VDG)‐entrapped and chondroitin sulfate (CS)‐modified LNPs (CS‐NPs/VDG) to repair capillarized LSECs and inhibit activated HSCs and constructed silybin (SIB)‐entrapped and glycyrrhetinic acid (GA)‐modified LNPs (GA‐NPs/SIB) to decrease ROS level, relieve hepatocytes damage, and promote cell regeneration.^158^ Despite these benefits, LNPs might be hampered by poor drug loading and biodistribution, resulting in their significant uptake by the liver and spleen. It is important to note that LNPs can vary depending on specific formulations and applications; complex formulation requires optimization of various parameters, needs proper storage and handling conditions, as well as limited drug loading capacity and potential immunogenicity will affect the value of clinical application.^153^, ^159^

#### 4.2.2 Polymeric nanoparticles

Polymeric NPs generally have ionizable amine groups, interacting with negatively charged nucleic acids containing phosphate groups via attractive coulombic forces and forming condensed nanosized structures termed polyplexes. And the most common forms are solid matrix systems, micelles, and polyplex NPs.^151^, ^160^ Unlike LNPs, polymeric NPs are composed of diverse chemical groups and use covalent and non‐covalent interactions, which provide additional opportunities for liver tissue aggregation^151^ (Figure 4). For example, Xing et al. developed vitamin A‐derivative (VA)‐decorated PEG‐PCL micelles to encapsulate the labile and hydrophobic camptothecin (CPT).^161^ CPT micelles not only specifically attack myofibroblastic HSCs (MF‐HSCs) and their glycolysis, effectively diminishing their myofibroblastic features and pronouncedly ameliorating liver injury and liver fibrosis.^161^ Therefore, polymeric NPs are ideal candidates for drug delivery because they are biodegradable, water soluble, biocompatible, biomimetic, stable during storage, and easily modified surface.^153^ However, polymeric NPs have the disadvantages of increased risk of particle aggregation and toxicity and limited clinical experience in the delivery of nucleic acids and drugs, and further exploration is still needed in the future.

### 4.3 Viral nanoparticles

Viral nanoparticles (VNPs) are sensitized to detect signals in the cellular environment, releasing their genome when they receive instructions, and they also have stable structures that can withstand environmental pressures and escape degradation.^171^ There are various VNPs used in preclinical and clinical applications, including adenoviruses, adeno‐associated viruses, and lentiviruses, and each has its advantages.^172^ And using VNPs in the liver cirrhosis treatment has achieved remarkable results. Approximately, 67% of gene therapy clinical trials worldwide use viruses as vectors to transport genetic materials.^171^ VNPs can achieve consistently high protein expression through carrying and delivering transgenes directly to target cells.^173^ For example, Chen et al. demonstrated that an adeno‐associated virus vector (serotype 6, AAV6) that carries a short‐hairpin (sh) RNA can target Nestin (AAV6‐shNestin) in HSCs to alleviate liver fibrosis.^174^ Mechanistically, knocking down Nestin in HSCs promoted caveolin 1 (Cav‐1)‐mediated TβRI degradation and downregulated TGF‐β signaling.^174^ For VNPs, the hollow inner cavity is also used as the biological carrier of drugs, vaccines, and molecular imaging probes.^175^, ^176^ In another study, Zhang et al. encapsulated Quercetin (QR) in hepatitis B core (HBc) protein nanocages (NCs) for targeted and imaging treatment of liver fibrosis.^177^ It can efficiently inhibit the proliferation and activation of HSCs and has great potential as an NIR fluorescent and magnetic resonance imaging contrast agent for liver fibrosis. However, VNPs also have some disadvantages that need to be considered, including host tropism and only infection or interaction with certain cell types or species, immunogenicity, safety concerns, complex, time‐consuming production, etc.^171^, ^172^

In end‐stage liver diseases, especially for patients with liver cirrhosis, the liver cannot support the growth of healthy tissue. Still, tissue engineering is an alternative to organ transplantation therapy, theoretically solving the major medical problem of insufficient donor organ sources.^178^ VNPs deliver specific genes to achieve liver tissue regeneration, which has been reported in many studies.^35^, ^179^ Genetic manipulation using VNPs provides a chance for the development of reprogramming and can directly convert the spleen into a liver‐like organ. For example, Liu et al. designed a three‐step strategy to transform the spleen into a liver‐like organ by directly reprogramming the splenic fibroblasts into hepatocytes in vivo.^180^ They used SiO_2_ NPs to activate splenic fibroblasts and generate more fibroblasts and ECM. Next, expressing three transcriptional factors (Foxa3, Gata4, and Hnf1a) through lentiviral vectors converts splenic fibroblasts into functional hepatocyte‐like cells. Finally, delivering tumor necrosis factor‐α/epidermal growth factor/hepatocyte growth factor increases the number of hepatocyte‐like cells to 8 × 10^6^ hepatocytes, and they have the same physiological function of liver.^180^ The significance of this technology is transforming a dispensable organ (including the spleen) into a native organ with specific physiological functions, focusing on reconstruction functions. It has more flexibility and efficiency.^178^

### 4.4 Cell membrane‐coated nanoparticles

Cell membrane‐coated nanoparticles (CNPs) are constructed from cell membrane or cell‐derived components, which make great strides in their applications for disease treatments.^173^ CNPs can be prepared by engineered cells; the synthesis typically comprises three distinct steps, including isolation of membrane from cells, NPs core preparation, and coating of the membrane onto the NPs core. This technology provides a simple method to introduce functionalized biological interface characteristics of cells into NPs without complex synthesis. And it is not easily copied by traditional synthesis technology.^173^ For example, Ma et al. constructed a biomimetic nanomaterial (named PM NPs), which encapsulates polydopamine with a macrophage membrane to target the diseased site of inflammation, simultaneously inhibiting inflammation and scavenge ROS, finally realizing the treatment of liver fibrosis.^181^ These NPs disrupt the cascade amplification and the vicious cycle between the liver microenvironment and provide a promising solution for liver fibrosis diseases related to inflammation and oxidative stress.

Some characteristics of natural cell membranes may increase the delivery ability of NPs, such as taking advantage of the site‐specific ligands to bind to surface receptors expressed on target cells specifically. However, various cells may express thousands of surface markers, which may not be enough to enhance the targeting effect.^182^ Using transgenic technology to achieve surface expression of natural cell membranes is highly desirable.^173^ For example, Xia et al. successfully established LX2 cells stably expressing tumor necrosis factor‐related apoptosis‐inducing ligand (TRAIL) (LX2‐TRAIL cells) via the transfection with a lentiviral vector expressing TRAIL‐ZsGreen and synthesized LX2‐TRAIL membrane‐coated poly(lactide‐co‐glycolide) (PLGA) NPs (TM‐NPs), which displayed synergetic effect to deplete aHSCs by inducing both apoptosis and quiescence.^183^ Besides, due to the natural source of the cell membrane, CNPs have superior biocompatibility and immune evasion capability. Meanwhile, CNPs may not be easily absorbed by the mononuclear phagocyte system and may have potential biological toxicity. However, it needs to be noted that they do not show high organ selectivities such as LNPs and polymeric NPs.^182^ Although the cell membrane provides additional stability, in certain circumstances (e.g., in blood or other liquids flowing in the body, NPs may be affected by mechanical forces such as shear force), the stability of the NPs may still be challenged.

### 4.5 Organelle nanoparticles

Organelle nanoparticles (ONPs) are nanomaterials whose surface is coated with substances that mimic or imitate the structure and function of specific organelles within the cells. Similar to CMPs, ONPs form a biomimetic structure that inherits the surface properties and functions of the organelle and imparts additional biological capabilities to the encapsulated NPs, such as low toxicity, low immunogenicity, high stability, high tissue tolerance, high biocompatibility, and ease of modification.^184^, ^185^ Different organelles from biological sources, including EVs, lipid droplets, lysosomes, and mitochondria, have been studied for drug delivery.^184^ Among various biomimetic drug delivery systems, the current research hotspots are organelle NPs dominated by EVs.^186^, ^187^ They have increasingly shown considerable promise as drug delivery systems, and the drug‐loading mode mainly includes endogenous techniques in which the EV‐producing cells also equip vesicles with cargoes (protein, drugs, and modified genes) and exogenous techniques in which cargoes are loaded into EVs after isolation.^188^ The endogenous techniques have a lower degree of complexity, but the drug‐loading process may compromise the integrity of EVs, and additional purification steps are required to remove uncoated drugs; the exogenous techniques have the advantage of retaining the integrity and function of EVs, but the operation is often complicated, the preparation cost is high, and the experimental period is long.^188^, ^189^

In treating liver cirrhosis, EV‐based nanomedical therapeutic platforms also play an extremely essential role. For example, Lin et al. fused the peptide (HSTP1) from a phage‐displayed peptide library with human umbilical cord mesenchymal stem cell (Huc‐MSCs)‐derived exosomes to enrich membrane protein (Lamp2b) through genetic engineering.^190^ The engineered exosomes (HSTP1‐Exos) are more efficiently internalized by HSC‐T6 cells and outperformed in promoting HSC‐T6 reversion to the quiescent phenotype. In vivo, results showed that HSTP1‐Exos could specifically target the aHSCs region after intravenous administration and enhance the therapeutic effect on liver fibrosis.^190^ In addition, EVs from different biological sources can play different therapeutic effects when used as delivery systems. The substantial progress has been made in the liver disease treatment by using NPs to deliver active substances of TCM.^158^, ^191^, ^192^, ^193^ It has been shown that Chinese herbal medicine‐derived EVs have similar pharmacological effects to those of the original plants, which can protect their components and achieve effective delivery to the target site.^194^ Therefore, plant‐derived EVs extracted from herbs as NPs transport plant‐specific active components and load proteins, nucleic acids, lipids, and other common ingredients (including drugs), achieving synergistic effects to treat liver cirrhosis. However, the research on plant‐derived EVs is still in its initial stage, and there is even less research on Chinese herbal medicines‐derived EVs. These NPs provide a new idea for treating liver cirrhosis due to the great design flexibility, enhanced functionality, and biocompatibility. However, the research is often restricted by using more advanced preparation technology and higher preparation requirements.

### 4.6 Nanozymes

Nanozymes are a cross‐product of many fields, including biological enzymes, nanomaterials, and surface catalysts.^176^, ^195^ The updated definition of nanozymes in 2021 is nanomaterials that catalyze the conversion of enzyme substrates to products and follow enzymatic kinetics (e.g., Michaelis–Menten) under physiologically relevant conditions, even though the molecular mechanisms of the reactions could differ between nanozymes and the corresponding enzymes.^196^ Currently, the main research focus on nanozymes includes two aspects: type 1 nanozymes (with nanomaterials used as immobilized catalysts or enzymes) and type 2 nanozymes (depending on the surface catalytic properties of inorganic nanomaterials).^197^ Many nanozymes have been discovered with remarkable enzyme‐like activities, which not only show behaviors such as natural enzymes but also modulate the ROS and nitrogen species (RONS) levels in biological cells^195^, ^197^ In addition, as nanomaterial, they can also deliver drugs to specific areas in the body.^195^ Given these new insights, nanozymes are proposed as an emerging vehicle that can be used to treat liver diseases. For example, Jing et al. presented a synergistic strategy for enhanced antifibrogenic therapy based on nilotinib (NIL)‐loaded hyaluronic acid (HA)‐coated Ag@Pt nanotriangular nanozymes (APNH NTs), which inherited superoxide dismutase and catalase activities.^198^ APNH NTs downregulate the expression of NADPH oxidase‐4 (NOX‐4) and inhibit HSC activation by efficiently eliminating intrahepatic ROS and producing oxygen to alleviate the hypoxic microenvironment further. They also synergistically remodel the microenvironment of liver fibrosis by releasing NIL to promote collagen depletion.^198^ In another study, Lu et al. developed a sequential delivery strategy for liver fibrosis treatment based on autophagy inhibitor carvedilol (CAR) and the HA‐modified star‐like Au nanozymes (Au NS@CAR‐HAs).^199^ CAR suppresses autolysosome generation by increasing autosome and lysosome pH values to inhibit HSC activation. Meanwhile, Au NS enhanced the ROS scavenging efficiency of hydrogen peroxides and superoxide and restrained peroxisome proliferator‐activated receptor β and c‐Jun N‐terminal kinase, thereby reducing the proliferation of HSCs.^199^ However, it is also necessary to assess the safety, efficacy, and feasibility of nanozymes for liver cirrhosis treatment before clinical implementation.

## 5 MULTITARGET NANOPARTICLES

Multitarget NPs are a type of nano‐delivery system with manifold functions designed to target multiple biological targets or pathological processes simultaneously. In recent years, multitarget codelivery systems or combinational delivery systems have provided an effective strategy for improving the liver microenvironment and enhancing the advantages of liver cirrhosis treatment.

**FIGURE 3:**
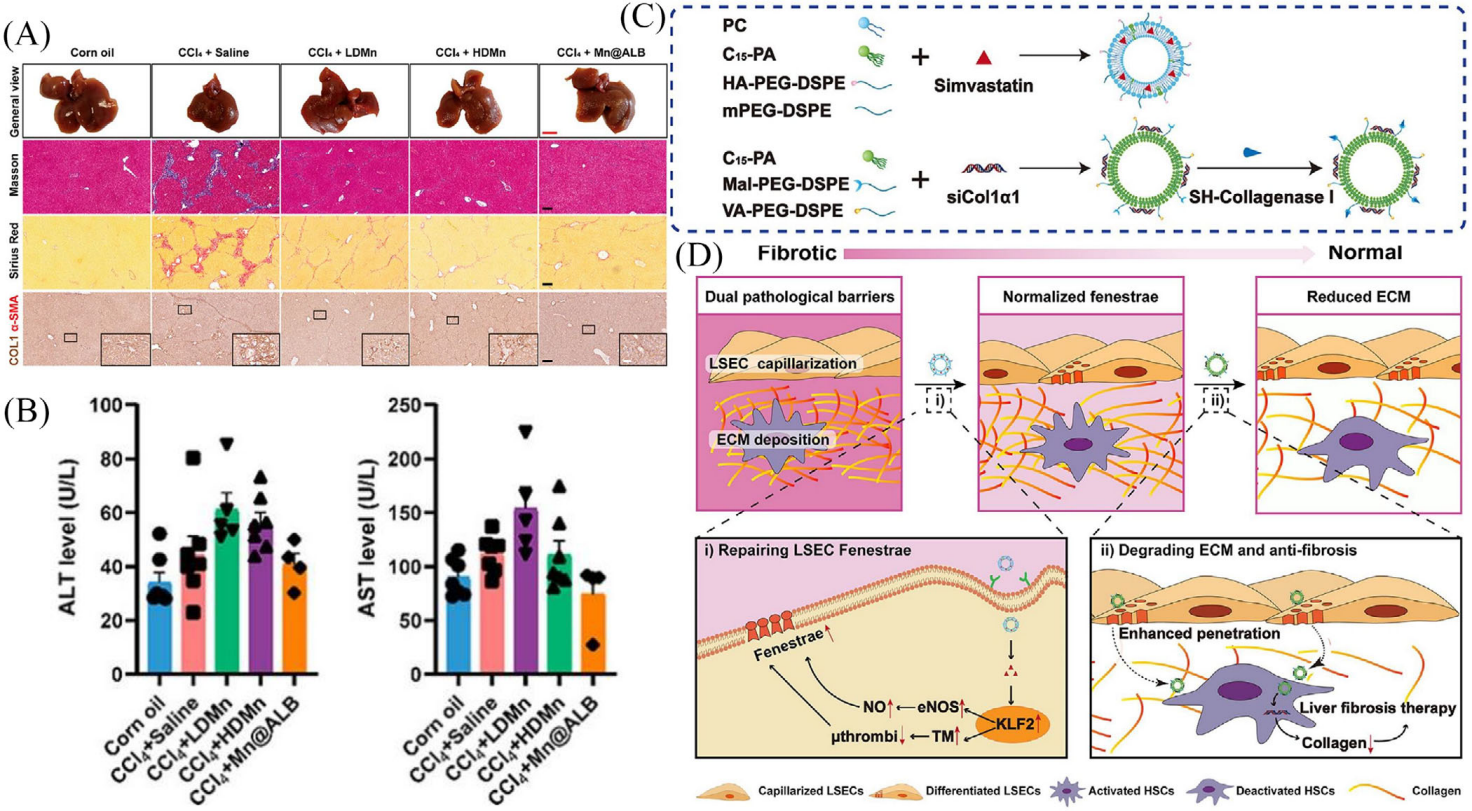
The advantages of multitarget nanoparticles for liver cirrhosis treatment. (A) General view, Masson's trichrome staining, Sirius Red staining, and immunohistochemical staining of α‐smooth muscle actin (α‐SMA) and COL1 in liver paraffin section slides showed both MnCl_2_ and Mn@ALB nanoparticles (NPs) therapy significantly reduced CCl_4_‐induced liver fibrosis. (B) Serum alanine aminotransferase (ALT) and aspartate transaminase (AST) (*n* = 4−7) demonstrated the improvement of liver function. Adapted from Ref.^203^ with permission, copyright the Author(s), 2024. (C) Preparation of hyaluronic acid (HA)‐NPs/simvastatin (SMV) and CV‐NPs/siCol1α1. (D) The expected effects of HA‐NPs/SMV and CV‐NPs/siCol1α1 in vivo on reversing liver fibrosis. Adapted from Ref.^211^ with permission, copyright American Chemical Society, 2024.

Capillarized LSECs mechanically restrict the transport of metabolites, drug molecules, and proteins between the blood and HSCs.^37^ The features that show loss of fenestrae and organized basement membrane deposition result in the apoptosis and necrosis of hepatocytes and the secretion of DAMPs.^36^ Moreover, they also change the intrahepatic hemodynamics and participate in the formation of PH.^200^ Therefore, the obstruction of tissue repair by capillarized LSECs needs to be considered in antifibrosis therapy. Previous studies have shown that transcytosis contributes to the transfer of molecules from the sinusoids to the space of Disse.^201^, ^202^ Therefore, Gu et al. used self‐assembly of Mn^2+^ and human serum albumin to obtain Mn@ALB NPs.^203^ First, LSECs have a strong capacity for albumin uptake, which is transferred across LSECs via transcytosis into the Disse space. Second, due to the high expression of secreted protein acidic and rich in cysteine, Mn@ALB NPs were specifically taken by activated HSCs. Finally, they realized the amelioration and recovery of liver fibrosis by releasing Mn^2+^ and activating the cGAS‐STING pathway to enhance the activation and cytotoxicity of NK cells (Figure 3A,B). In another way, reprogramming LSECs into the differentiated phenotype with fenestration and non‐septum pores can promote the penetration of NPs into the space of Disse in liver fibrosis tissue.^204^, ^205^ Based on the previous research, Li et al. devised a combined therapeutic approach for the liver cirrhosis treatment, wherein the soluble guanylate cyclase stimulator (riociguat) was utilized to reverse capillarized LSECs and maintain their normal porosity. This facilitated the transport of peptide NPs (IGNP‐JQ1) through the liver sinusoid endothelial wall, leading to enhanced accumulation of IGNP‐JQ1 in the space of Disse. Subsequently, IGNP‐JQ1 was selectively taken up by activated HSCs, inhibiting their proliferation and reducing collagen deposition in the liver, thereby mitigating liver fibrosis.^206^

Liver fibrosis caused by persistent injury is accompanied by excessive ECM, which gradually replaces liver parenchyma and hinders the delivery of NPs to HSCs.^37^ Advanced glycation end products mediate high levels of crosslinking in ECM, which are a hallmark of liver cirrhosis and significantly change the structure and mechanical properties of collagenous fibers to form an abnormal liver microenvironment and regulate the phenotype and function of cells.^207^, ^208^ The ECM degradation is facilitated by a cell therapy strategy (ECM‐degrading LSECs are screened using a cell‐mediated ECM‐degradation screening system in vitro), which can be used in liver cirrhosis treatment.^209^ In addition, Fan et al. prepared a collagenase I and retinol co‐decorated polymeric micelle that possesses nanodrill‐like and HSCs‐targeting functions based on poly‐(lactic‐co‐glycolic)‐b‐poly(ethylene glycol)‐maleimide (PLGA‐PEG‐Mal) (named CRM).^210^ Upon encountering the collagen I barrier, CRM exerted a nanodrill‐like function, efficiently degrading pericellular collagen I and showing greater uptake by human HSCs than other micelle formulations. CRM loaded with nilotinib reduces the expression of metallopeptidase inhibitor TIMP‐1, and in turn, enhances collagen I degradation, thereby exerting therapeutic effects on liver fibrosis.

It is almost neglected that the complex microenvironment of liver cirrhosis significantly impedes drug delivery to activated HSCs, especially the capillarized LSEC barrier and the deposited ECM barrier.^24^, ^37^ Therefore, breaking through capillarized LSEC barrier and deposited ECM barrier at the same time to achieve drug delivery can improve the efficiency of drug delivery within the liver, making it easier for drugs to reach the diseased areas and restore liver function. For example, Zhang et al. developed a strategy for regulating pathological barriers using a sequential nanopenetrator system to achieve the deep delivery and precise targeting of HSCs for liver fibrosis therapy^211^ (Figure 3C,D). For the first barrier, LSECs‐targeting and fenestrae‐repairing NPs (HA‐NPs/SMV) were designed based on the modification with HA and the loading of simvastatin (SMV). For the second barrier, collagenase I‐ and vitamin A‐codecorated NPs with collagen‐ablating and HSC‐targeting functions (CV‐NPs/siCol1α1) were prepared to deliver siCol1α1 with the goal of inhibiting collagen generation and HSC activation. Upon encountering the capillarized LSEC barrier, HA‐NPs/SMV rapidly released SMV and exerted a fenestrae‐repairing effect, which allowed more CV‐NPs/siCol1α1 to enter the space of Disse to degrade deposited collagen and ultimately achieve greater accumulation in activated HSCs.

## 6 NANOMEDICINE‒BIOLOGICAL ENVIRONMENT INTERACTIONS

**FIGURE 4:**
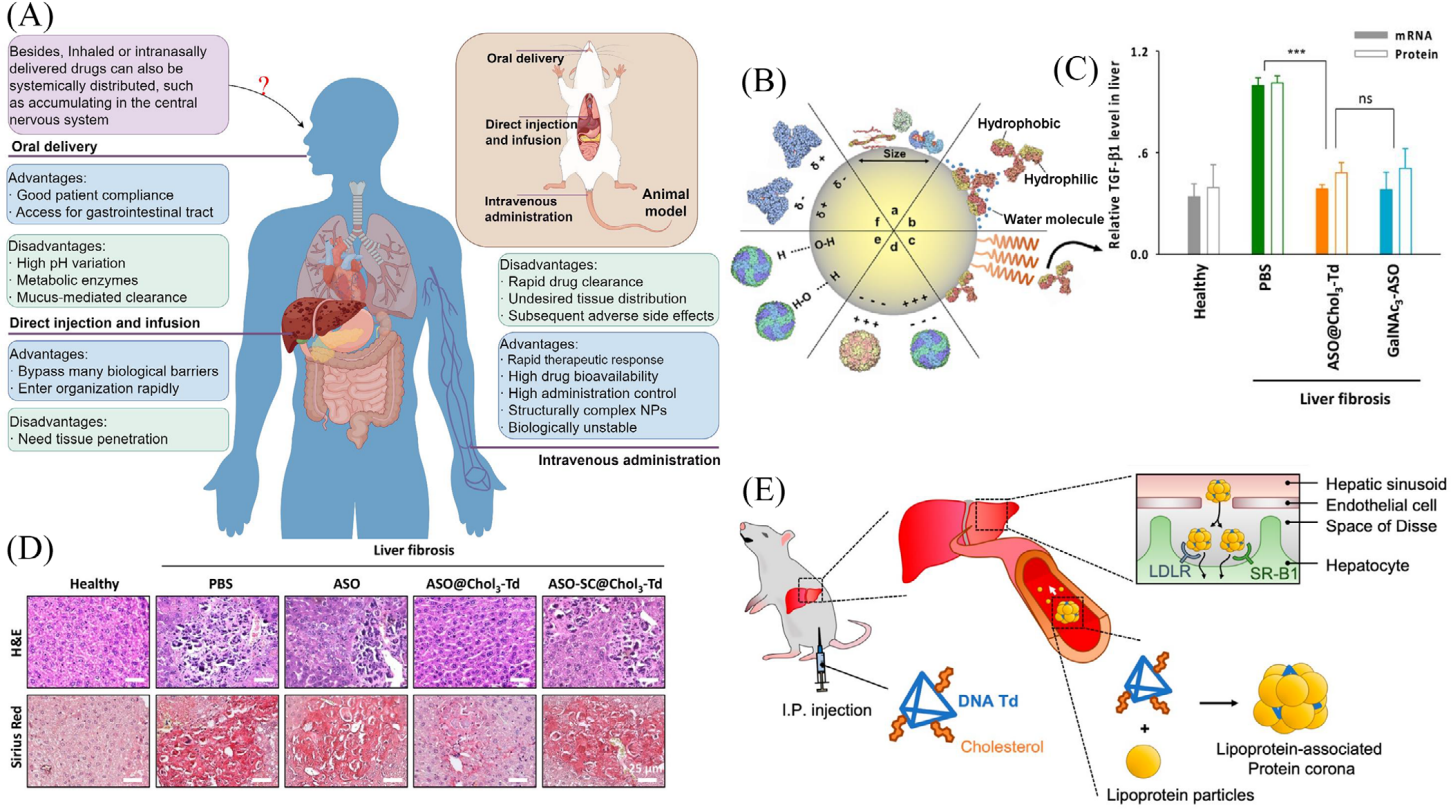
The interactions between nanomedicine and biological environment. (A) Advantages and disadvantages of different administration routes for nanoparticles (NPs). This figure was generated using FigDraw (https://www.figdraw.com). (B) The interactions between NPs and proteins in the biological environment after their encounter. Adapted from Ref.^229^ with permission, copyright Elsevier, 2023. (C) Quantitative real‐time PCR (qRT‐PCR) analysis showed that ASO@Chol_3_‐Td and GalNAc_3_‐ASO substantially decreased the transforming growth factor‐beta 1 (TGF‐β1) mRNA and protein levels. (D) Images of liver sections stained with hematoxylin and eosin (H&E) and Sirius red showed that acute necrosis and inflammatory cells around the portal vein generated by chronic injury were substantially decreased by ASO@Chol_3_‐Td. (E) Protein corona (PC) was formed with intraperitoneally injected Chol_3_‐Td to steer NPs to hepatocytes. Adapted from Ref.^220^ with permission, copyright American Chemical Society, 2022. ASO, antisense oligonucleotide; Chol_3_‐Td, DNA tetrahedron with trivalent cholesterol conjugation; GalNAc_3_, liver‐targeting ligand, trivalent N‐acetylgalactosamine.

Despite the potentially wide‐ranging applications of NPs in liver cirrhosis treatment, the performances of clinical trials are not ideal. The huge gap is in part due to the absent understanding of the nanomedicine‒biological environment interactions in patients. First, each route of administration leads to a distinct drug distribution and distinct patterns of adverse effects,^212^ as shown in Figure 4A. Besides, the arrival of NPs in the cells is an extremely complicated process, shaped by many factors, including unique physicochemical characteristics of NPs, protein‒NPs interactions, and subsequent agglomeration, diffusion, and sedimentation.^213^ Under the influence of the biological environment, NPs will transform into substances such as cofactors and ions or release active substances to exert the function of biological regulatory effects in vivo.^214^

Once it enters the bloodstream, NPs inevitably interact with biomolecules, resulting in the formation of PCs on their surface.^215^, ^216^ Proteins adsorb onto the surface of NPs to form the PCs, which leads to an increase in the size of the NPs and changes in the surface chemistry, affecting the various crucial processes of the NPs in vivo, including the stability, targeting, translocation, biotransformation, and clearance of NPs in the body^216^ (Figure 4B). However, high levels of cholesterol in serum could reduce the thickness of PCs on NPs through remodeling the protein abundance, and it may be associated with the Vroman effect.^215^, ^217^ This effect regulates and optimizes protein adsorption of the NPs’ surface to improve their stability and biocompatibility. For example, previous studies have shown that oligonucleotide therapeutics such as siRNAs and ASOs are efficiently delivered to hepatocytes by cholesterol conjugation to treat liver diseases.^218^, ^219^ Therefore, Kim et al. prepared a DNA tetrahedron with three cholesterol conjugations (Chol3‐Td), and they can deliver ASO‐targeting TGF‐β1 mRNA to treat liver fibrosis.^220^ The potency of ASO@Chol3‐Td was comparable to that of ASO conjugated with the clinically approved liver‐targeting ligand, trivalent N‐acetylgalactosamine (GalNAc3) (Figure 4C‒E). This study suggested that controlled seeding of the PCs on nanomaterials provides a way to steer NPs into the target area. However, improper protein adsorption might trigger immune responses, leading to the removal of NPs or other adverse reactions.^216^ Besides, due to individual biological differences, including age, gender, race, and health status, as well as various biomolecules in the blood, the same NPs could form different PCs.^221^, ^222^, ^223^

The unique organ structures and blood flow characteristics more easily promote the NPs‒liver interactions, and it may also cause more serious adverse effects.^224^ For example, excessive accumulation of NPs in the liver may lead to hepatotoxic reactions. It should be highlighted that the specific uptake and response of different cell types are essential to recognize the effect of nanomedicine therapy.^224^ NPs first encounter KCs when entering the hepatic sinuses and are the notable populations of macrophages involved in the sequestration of NPs. NPs larger than 100 nm and with a negative charge or hydrophilicity are easily taken up by KCs via phagocytosis.^225^ LSECs are specialized scavenger endothelial cells and are regarded as one of the highest endocytic activities of any cell type in the body.^226^ NPs less than 200 nm in diameter, with a negative charge or hydrophobicity, tend to be taken up by LSECs through clathrin‐mediated endocytosis at a high exposure dose or for a long time. Besides, NPs less than 50 nm in diameter and hydrophilic are captured by HSCs, and mall NPs with a positive charge or hydrophilicity are preferentially taken up by hepatocytes through clathrin‐mediated endocytosis.^225^ Among them, glutathione‐mediated biotransformation is strongly size dependent (<100 nm), and the smaller NPs can more easily undergo glutathione‐mediated biotransformation when the size is reduced to below 6 nm.^225^ In the process, NPs induce a large variety of intracellular responses, depending on their physicochemical properties, intracellular concentration, duration of contact time, subcellular distribution, and interactions with biological molecules.^227^

More attention should be paid to the fate, biokinetic behavior, and bioavailability of these compounds in vivo, and the underlying mechanism of the medicinal efficacy when designing therapeutic NPs.^214^ This is closely related to optimizing the release rate and distribution of drugs in the body, avoiding excessive accumulation or rapid metabolism of drugs to enhance treatment efficacy and reduce side effects. With the significant development of nanomedicine‒biological environment interactions, many engineering strategies have been put forward, such as constrain size, external stimulus, active transport, improved blood circulation, biological distribution, and tissue accessibility to optimize the design of NPs.^228^ By comprehensively applying these engineering strategies, the stability, targeting, and biocompatibility of NPs in biological environments can be improved. However, the process is still not deeply understood, and more exploration is still needed in the future.

## 7 CONCLUSIONS AND PERSPECTIVES

Liver cirrhosis is an important cause of morbidity and mortality in people with chronic liver disease worldwide, and developing a standard treatment for liver cirrhosis is currently an urgent need. The occurrence and development of liver cirrhosis are often accompanied by abnormal changes in the liver microenvironment, which involves multiple types of cells (including HSCs, LSECs, hepatocytes, KCs, and additional immune cell populations) and complex processes (including interaction with each other). In this review, we highlighted the crucial role of maintaining the steady state of the liver microenvironment and described the molecular mechanisms and different intervention strategies for liver cirrhosis; among them, nanomedicine has enormous potential in realizing the reconstruction of liver tissue function by improving the disorders of the liver microenvironment and provides a new idea for the research of therapeutic drugs. Besides, the interactions between nanomedicine and the biological environment should also be taken seriously due to it is associated with the stability, targeting, biocompatibility of NPs in biological environments, etc.

Although the treatment of liver cirrhosis has achieved excellent results, further exploration is still needed in the research of therapeutic drugs. First, elucidating the essential roles and dynamic changes of key cell types in the liver microenvironment is crucial for deeply understanding the molecular mechanisms of liver cirrhosis. It is also necessary to note that liver fibrosis is a common feature of all types of chronic liver injury. There appear to be unique aspects to different etiologies‐driven liver fibrosis. Besides, we still need to use the existing technologies (such as single‐cell technologies) to deeply explore the subtypes and potential functions of different cell types and better clarify the molecular mechanism of liver cirrhosis development. With the rapid growth of nanotechnology, except conventional inorganic and organic NPs, various biological NPs are more widely used for drug and gene delivery. They have different advantages (e.g., low immunogenicity, high stability, and high biocompatibility) and may become the focus of drug research in the future. Therefore, selecting suitable materials is a critical first step in the design of organ‐targeted delivery systems. However, research on these NPs has only been limited to animal experiments, and the differences between animal models and human physiological structures are important reasons for the poor clinical efficacy of these drugs. Finally, the nanomedicine‒biological environment interaction is a frequently neglect issue in most studies. Although the important influencing factors (such as PCs) are being studied, most of them are still elusive. Therefore, more efforts are needed to reveal the molecular regulation mechanism and better understand the fate of NPs with different characteristics in vivo. Answering these questions may provide new insight into liver cirrhosis treatment.

In conclusion, current therapeutic strategies, especially nanomedicine, provide more opportunities for liver cirrhosis treatment, but they also face more challenges. In the future, we need to combine various factors to consider these issues and achieve the expected effects while minimizing the adverse effects.

## AUTHOR CONTRIBUTIONS

Z.D. contributed to the conception of the study and wrote the initial draft. Y.W. reviewed the manuscript and provided suggestions for revision. W.J. helped perform the analysis with constructive discussions. All authors read and approved the final manuscript.

## CONFLICT OF INTEREST STATEMENT

The authors declared they have no conflicts of interest.

## ETHICS STATEMENT

Not applicable.

## Data Availability

Not applicable.
